# Robust visual cortex evoked potentials (VEP) in Gnat1 and Gnat2 knockout mice

**DOI:** 10.3389/fncel.2022.1090037

**Published:** 2022-12-20

**Authors:** Michael D. Flood, Hannah L. B. Veloz, Samer Hattar, Joao L. Carvalho-de-Souza

**Affiliations:** ^1^Department of Anesthesiology, College of Medicine, The University of Arizona, Tucson, AZ, United States; ^2^Section on Light and Circadian Rhythms (SLCR), National Institute of Mental Health, Bethesda, MD, United States; ^3^Department of Physiology, College of Medicine, The University of Arizona, Tucson, AZ, United States; ^4^Department of Ophthalmology and Vision Science, College of Medicine, The University of Arizona, Tucson, AZ, United States; ^5^BIO5 Institute, The University of Arizona, Tucson, AZ, United States

**Keywords:** retina, phototransduction, transducin, ipRGCs, melanopsin, visual evoked potential, primary visual cortex, vision

## Abstract

Intrinsically photosensitive retinal ganglion cells (ipRGCs) express the photopigment melanopsin, imparting to themselves the ability to respond to light in the absence of input from rod or cone photoreceptors. Since their discovery ipRGCs have been found to play a significant role in non-image-forming aspects of vision, including circadian photoentrainment, neuroendocrine regulation, and pupillary control. In the past decade it has become increasingly clear that some ipRGCs also contribute directly to pattern-forming vision, the ability to discriminate shapes and objects. However, the degree to which melanopsin-mediated phototransduction, versus that of rods and cones, contributes to this function is still largely unknown. Earlier attempts to quantify this contribution have relied on genetic knockout models that target key phototransductive proteins in rod and cone photoreceptors, ideally to isolate melanopsin-mediated responses. In this study we used the Gnat1^–/–^; Gnat2^cpfl3/cpfl3^ mouse model, which have global knockouts for the rod and cone α-transducin proteins. These genetic modifications completely abolish rod and cone photoresponses under light-adapted conditions, locking these cells into a “dark” state. We recorded visually evoked potentials in these animals and found that they still showed robust light responses, albeit with reduced light sensitivity, with similar magnitudes to control mice. These responses had characteristics that were in line with a melanopsin-mediated signal, including delayed kinetics and increased saturability. Additionally, we recorded electroretinograms in a sub-sample of these mice and were unable to find any characteristic waveform related the activation of photoreceptors or second-order retinal neurons, suggesting ipRGCs as the origin of light responses. Our results show a profound ability for melanopsin phototransduction to directly contribute to the primary pattern-forming visual pathway.

## Introduction

When intrinsically photosensitive ganglion cells were first discovered they were thought to consist of a single population of retinal neurons, now known as M1 ganglion cells (Berson et al., 2002; Hattar et al., 2002). Interestingly, these cells only sparsely project to the dorsal lateral geniculate nucleus, which transmits retinal information to the primary visual cortex, but instead convey the majority of their information to a variety of nuclei outside of the classical visual pathway (Hattar et al., 2006; Li and Schmidt, 2018). It was quickly determined that these ipRGCs played important roles in non-imaging-forming aspects of vision, including circadian photoentrainment, neuroendocrine regulation, and the pupillary light response (Altimus et al., 2008; Güler et al., 2008; Hatori et al., 2008; LeGates et al., 2012). Because melanopsin was found to be the photopigment responsible for the intrinsic photosensitivity of these cells, it was assumed that its functional significance was restricted to these non-image forming circuits. However, with advancements in immunohistochemical detection techniques melanopsin was found to be expressed in at least 6 different classes (Estevez et al., 2012; Renna et al., 2015; Quattrochi et al., 2019) of retinal ganglion cell (designated as M1-6), some of which project to regions of the brain involved in image formation (Ecker et al., 2010; Aranda and Schmidt, 2021). This discovery established the idea that, by definition, some ipRGCs are involved in the generation of pattern-forming visual perception.

Because all ipRGCs receive input from rod and cone photoreceptors pathways in addition to their intrinsic melanopsin photoexcitability (Zhao et al., 2014), an ongoing question is to what degree melanopsin signaling itself contributes to pattern-forming vision. Naturally this is a fairly difficult question to answer, as the separation of rod, cone, and melanopsin contributions to retinal signaling is not a straightforward enterprise (Aranda and Schmidt, 2021). Several studies in the past few years have attempted to answer this question by employing a variety of approaches. These include the selective knockout of rod and cone or melanopsin phototransduction (Brown et al., 2010; Ecker et al., 2010; Schmidt et al., 2014; Procyk et al., 2015), the ablation of specific cell populations (Güler et al., 2008; Schmidt et al., 2014; Hughes et al., 2016; Eleftheriou et al., 2020), and the use of dreadd agonists (Milosavljevic et al., 2016; Sonoda et al., 2018) or silent-substitution stimuli (Allen et al., 2014; Davis et al., 2015) to selectively activate melanopsin over rods and cones. Each of these approaches have different advantages and disadvantages, but it is likely that all are required to gain a complete and thorough understanding of melanopsin’s role in pattern-forming vision.

In this study we sought to determine the sufficiency of melanopsin expression alone for generating cortical activity in pattern-forming centers of the brain. Previous attempts to do so have relied on the use of Gnat1^–/–^; Cnga3^–/–^ double knockout mice (Ecker et al., 2010), in which the major pathways for rod and cone phototransduction have been ablated, or mouse models of photoreceptor degeneration in which large numbers of photoreceptors die off (Procyk et al., 2015). Both found evidence for major light-induced activity in the primary pattern-forming visual pathway (either in the dLGN or primary visual cortex), presumably from the activation of ipRGCs. However, in the former case of Gnat1^–/–^; Cnga3^–/–^ knockouts, some alternative pathway for rod signaling *via* cone photoreceptors, locked in “light” state by Cnga3 disruption, seems to be retained (Allen et al., 2010), providing a confounding mechanism for the observed photosensitivity. Similarly, in Rd1 mouse models it has been demonstrated that a small portion of cone photoreceptors in the peripheral retina survive into P90 (Lin et al., 2009) and beyond (Amamoto et al., 2022), and could explain residual photosensitivity in these animals. To address these potentially confounding issues, here we employed the Gnat1^–/–^, Gnat2^cpfl3/cpfl3^ mouse model, in which the genes encoding the α-subunit of both rod- and cone-transducin have been knocked out and mutated, respectively. Without both α-transducins, phototransduction *via* the rod or cone pathway should be impossible under light-adapted conditions, leaving melanopsin-mediated signaling as the only remaining mechanism for visual perception.

## Materials and methods

### Animals

Experiments were performed in accordance with rules and regulations of the National Institutes of Health guidelines for research animals, as approved by The University of Arizona Institutional Animal Care and Use Committee. A total of 13 C57BL/6J mice (Jackson Laboratories, Bar Harbor, ME, USA) were employed as controls in this study. These animals consisted of 5 males and 8 females and were between 15 and 30 weeks of age. A breeding pair of Gnat1^–/–^; Gnat2^cpfl3/cpfl3^ mice bred on a C57BL/6J background was kindly provided by Dr. Samer Hattar and propagated to form our own colony. In these animals the gene encoding the α subunit of rod transducin, *gnat1*, has been knocked out (Calvert et al., 2000) and the *gnat2* gene, encoding the α subunit of cone transducin, harbors a naturally occurring missense point mutation (c.598G > A; p.D200N) that completely extinguish cone-mediated responses from 9 weeks of age on (Chang et al., 2006; Ronning et al., 2018). A total of 11 male and 8 female Gnat1^–/–^; Gnat2^cpfl3/cpfl3^ mice, henceforth referred to as double knockout (DKO) mice, between the ages of 9–28 weeks were utilized for this present work. All animals were housed on a 12:12 light:dark cycle and provided with National Institutes of Health-31 rodent diet food and water *ad libitum*.

### VEPs and ERGs

**FIGURE 1:**
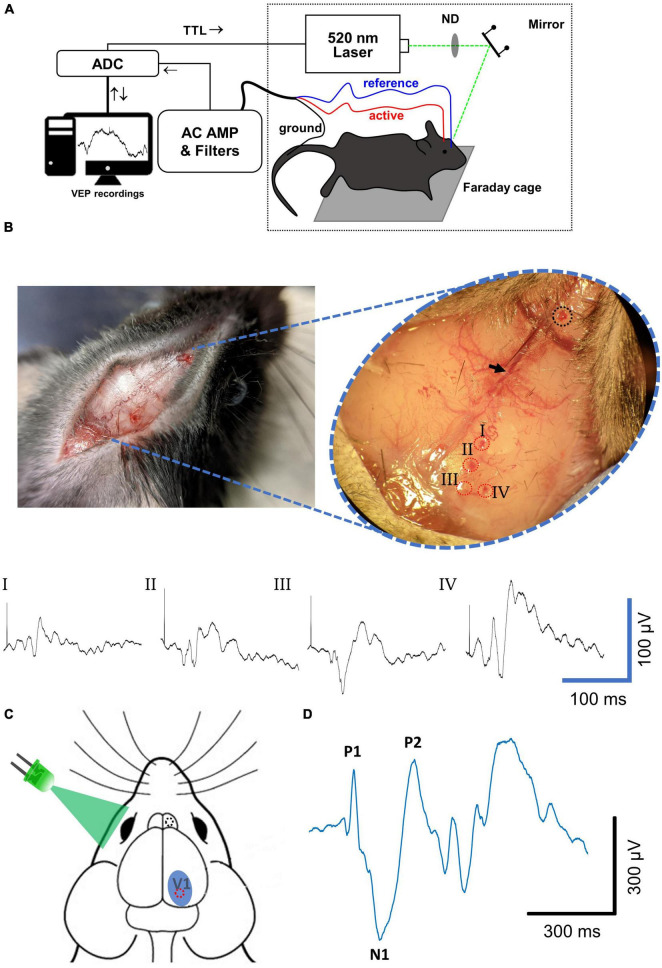
Visually evoked potential recording methodology. **(A)** Diagram of experimental setup for VEP recordings. **(B)** Characterization of spatial variability of VEP. (Left) A midsagittal incision through the dermis followed by dilation of connective tissue with a scalpel exposes the major cranial landmarks used for targeting of drill sites. (Inset) Locations used for VEP recordings are defined based on their relative distances from the bregma (black arrow) and the sagittal suture. As an exploratory measure, four active electrode drill sites (I–IV, red dotted circles) were drilled with a reference electrode site drilled rostral to the bregma (dotted black circle) and VEPs using each active electrode were recorded in the same experimental session. Resulting averaged VEP signals elicited with a 0.1 ms 520 nm laser stimulus are shown below. The site that resulted in the most robust VEP signal with most stereotypical waveform was site IV. **(C)** Based on the results in panel **(B)**, for all LED and saturating stimuli experiments the active electrode drill site (red circle) was drilled ∼3.6 mm caudal to the bregma and 1.7 mm lateral to the midsagittal plane that equates to site IV. The reference electrode drill site (black circle) was drilled 1–2 mm rostral to the bregma and ∼0.3 mm lateral to the midsagittal plane. The active electrode site chosen lies roughly in the center of the primary visual cortex (V1). The reference electrode site was positioned to lie over the olfactory bulb. LED and laser stimuli were applied to the contralateral (left) eye to evoke potentials in the right cortex. **(D)** An example VEP signal collected after averaging 20 sweeps. A typical waveform included a first positive deflection (P1) followed by a first negative deflection (N1) followed by a second positive deflection (P2), from baseline. Mouse head schematic in **(C)** was sourced and modified with permission from https://scidraw.io.; http://doi.org/10.5281/zenodo.3925903.

On the day of each experiment, mice were brought in cages from animal facility and placed under room lighting for at least 1 h to adapt to ambient conditions (∼375 μW/cm^2^, white light). Animals were anesthetized by intraperitoneal injections of 100 mg/kg ketamine + 16 mg/kg xylazine dissolved in normal saline. Degree of sedation was continuously monitored and maintained as needed by intraperitoneal injection with 50 mg/kg ketamine. After induction of anesthesia, heads were shaved, and dermis was separated cleanly with a mid-sagittal incision to expose the skull. Two holes were carefully drilled through the skull with a 30-gauge needle at a site ∼2.6 mm rostral to the bregma and 0.4 mm lateral to the midsagittal plane, and at a site ∼3.5 mm caudal to the bregma and ∼1.7 mm lateral to the midsagittal plane for a reference and active electrode, respectively (see Figure 1). The active electrode site was chosen to target the activity of right primary visual cortex. The contralateral (left) eye of each animal was dilated with a drop of 1% tropicamide ophthalmic solution (Bausch and Lomb, Bridgewater, NJ, USA) at least 5 min before any response was recorded. GenTeal Tears lubricating eye drops (Alcon Labs, Fort Worth, TX, USA) were applied as needed to prevent eyes from drying out during experiment. After skull preparation, animal was moved to a faraday cage and prepared for VEP recordings. Head stabilization was accomplished *via* a bite plate, a steel needle was inserted into the tail to act as signal ground and active and reference steel electrodes were maneuvered *via* micromanipulator into their respective drill holes to rest gently on top of the cortex. Signals were amplified with a Dam80 AC differential amplifier (World Precision Instruments, Sarasota, FL, USA) at a gain of 1,000× and bandpass-filtered between 10 Hz and 3 kHz with single-pole butterworth filters. For ERG recordings everything was kept the same except that the active electrode was instead manipulated to rest gently on top of the cornea and signals were bandpass filtered between 0.1 Hz and 0.3 kHz. All signals were digitized at 50 kHz with a 16-bit A/D converter (USB-1604, Measurement Computing, Norton, MA, USA) using in-house software (Gpatch64MC). This software also controlled the timing of all light stimuli used. During all steps of experimentation after anesthesia induction, animal body temperature was maintained at 37°C by a homeothermic monitoring system (Harvard Apparatus, Holliston, MA, USA).

### Light stimuli

The light stimuli employed in this study were generated with three different light sources: a 520 nm 500 mW semiconductor laser (Civil Laser, Zhejiang, China), an 808 nm 1 W semiconductor laser (Civil Laser, Zhejiang, China), and a 530 nm high power mounted LED (ML530L4, ThorLabs, Newton, NJ, USA). Each laser output was reflected using a system of optical mirrors (FMP1, Thor Labs, Newton, NJ, USA) which could be finely adjusted to align the laser spot with the left eye of each mouse. An infrared viewer (IRV1-1700 Newport, Irvine, CA, USA) was employed to align the 808 nm laser onto the mouse eye. The mounted LED was pointed directly at the mouse head, and its homogenized beam illuminated the entire left side of the face. Unattenuated power of each light source measured at the animal eye level was 2960, 8,000, and 44.3 mW/cm^2^ for the 520 nm laser, 808 nm laser and 530 nm LED, respectively. Laser stimuli total energy was varied by controlling the stimuli durations (0.01–1 ms), while LED energy was controlled by power, both by setting LED power output on its LEDD1B driver (0–100%, ThorLabs, Newport, NJ, USA) and by different combinations of neutral density filters in the light path (ND0–ND3). For recording of LED-only responses, VEPs were recorded from lowest to highest flash intensity in sequential order to minimize any effects of bleaching or adaptation. For each light intensity, twenty responses were recorded with an inter-stimulus-interval of 5 s. For saturating light stimuli, laser pulses were applied halfway through a 1 s LED flash of unattenuated light at maximum power. Between 22 and 30 responses were recorded for laser stimuli of 0.6 ms duration, with an inter-stimulus interval of 10 s. Before each set of stimuli, mice were given a recovery period of 2 min during which no light stimuli were applied. For laser-only stimuli, responses were collected with an inter-stimulus interval of 2 s, but the total number of responses was minimized to prevent retinal damage. These were recorded at the start of an experiment to ensure that each animal (both control and DKO) had a measurable VEP. Typically, the averaging of only 3–7 responses was enough to provide high enough signal-to-noise ratio for assessment of laser-induced VEP parameters. ERGs were recorded with similar laser stimuli, but at the end of an experiment after VEP recordings had been completed. Photon flux values for LED and laser power delivered to the eye were calculated assuming the total collecting area of a fully dilated C57BL/6-strain pupil of 3.2 mm^2^ and a total retina surface area of 17.8 mm^2^ (Lyubarsky et al., 2004).

### Data analysis

**FIGURE 2:**
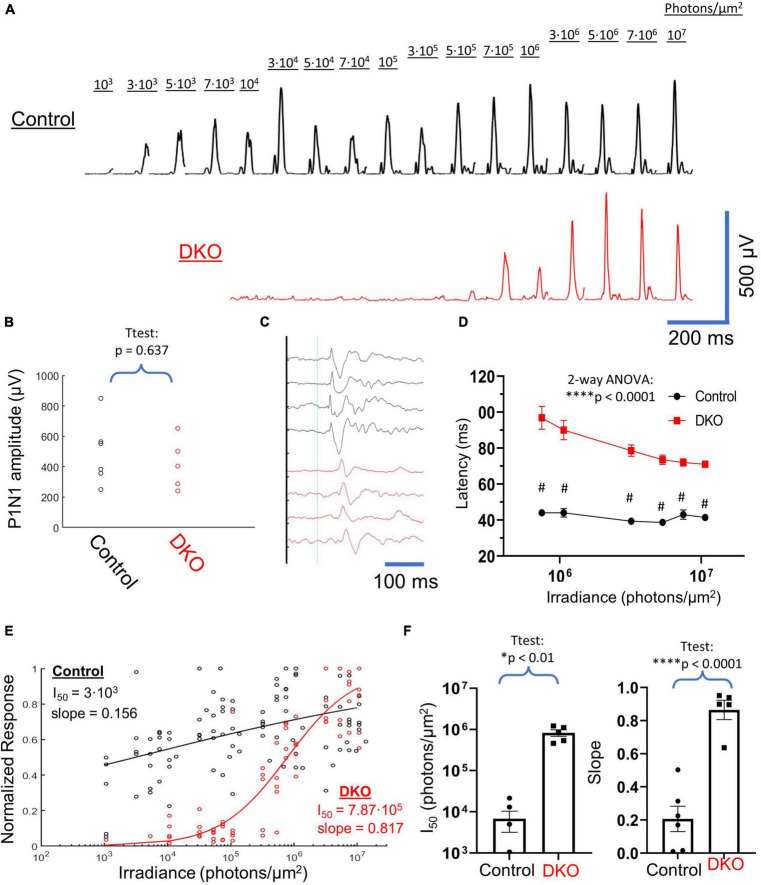
VEPs in DKO mice are consistent with a melanopsin-mediated mechanism. **(A)** Example averaged VEP responses for a control mouse (Top, black) and a DKO mouse (bottom, red) across 4 log units of light stimulus intensity. The photon irradiance delivered to the eye during each stimulus is shown. DKO responses only start to become apparent around 2 log units after threshold responses in controls. **(B)** Scatterplot of un-normalized P1N1 amplitudes at the highest 50-ms 532 nm LED flash (10^7^ photons/μm^2^) intensity used. There was no significant difference in this maximal response amplitude between control and DKO animals. **(C)** Averaged responses (normalized to P1 amplitude) at maximal light stimulus from 4 control (black) and 4 DKO (red) mice, showing the delayed kinetics of DKO VEPs. Thin blue line represents stimulus onset. **(D)** Average ±SEM latencies of P1 peak in control (black) and DKO (red) mice across the highest six intensities used (which were the only intensities that provided consistent VEPs in DKO mice). At all of these intensities, DKO VEP latencies were significantly delayed compared to controls. **(E)** Scatterplot of normalized VEP power vs. irradiance for all control (black) and DKO (red) data. Representative naka-rushton fits to each dataset, along with accompanying fit parameters, are shown. **(F)** Extracted naka-rushton parameters from individual fits to each mouse. DKO mice had both *I*_50_ values and slopes that were significantly larger than controls. Data is plotted individually (filled circles and squares as indicated) and bars are averages ± SEM. *Level of significant difference *via t*-test or two-way ANOVA main-effects. *p < 0.01 and ****p < 0.0001. ^#^Sidak multiple comparisons test *p* < 0.0001.

All VEP responses were first analyzed by custom MatLab (Mathworks, Carlsbad, CA, USA) scripts. Cortical responses for each stimulus were averaged to extract a waveform with discernable positive P1, negative N1, and positive P2 components (Ridder and Nusinowitz, 2006). Certain traces were excluded from the average if their signal-to-noise ratio was less than 1. VEP latencies were calculated as the time between the onset of the stimulus and the peak of the P1 component. VEP amplitudes were calculated as the absolute difference in voltage between the peaks of the P1 and N1 components. For power calculations, average traces were squared and integrated in time over the period from stimulus onset to 400 ms after. All power values were normalized across LED intensities to the maximum power value recorded for each individual mouse. Normalized power-intensity curves were fit both combined (Figure 2E) and individually (Figure 2F) to naka-rushton models according to the equation:


$$R=I^{slope}/(I^{slope}+I_{50}{}^{slope})$$

Where R is the normalized response, *I* is flash intensity, *I*_50_ is the flash intensity needed to elicit a half-maximal response and slope is a dimensionless unit that informs us of the degree of response heterogeneity (Evans et al., 1993). Tests for differences between control and DKO responses were performed in GraphPad Prism 9 (GraphPad Software, San Diego, CA, USA) and employed simple unpaired *t*-tests or two-way RM ANOVAs as appropriate. Pairwise differences were assessed *via* the Sidak-Dunn method for multiple comparisons. For all statistical tests, an α value of 0.05 was used.

### Immunohistochemistry

Immediately after electrophysiological recordings were completed, animals were sacrificed and their eyes were removed and drop-fixed in 4% paraformaldehyde in phosphate-buffered-saline (PBS) (Ref: J19943-K2, Thermo Fisher Scientific, Waltham, MA, USA) for 24 h. After fixation eyes were washed three times with PBS, processed, embedded in paraffin, and sectioned at a thickness of 5 μm. To begin immunohistochemistry (IHC), slides were first deparaffinized with two consecutive 5-min washes in Xylene, then gradually rehydrated with 3-min graded ethanol washes. Antigen retrieval was next performed by covering the slides in citrate buffer (1X) in a heat-safe container and heating this container under high pressure for 15 min in a commercial pressure cooker. After 15 min, the container was removed from the pressure cooker and allowed to gradually cool to room temperature. After cooling slides were quickly rinsed with water and PBS, followed by a 20-min permeabilization with 0.2% Triton X-100 (Sigma-Aldrich, St. Louis, MO, USA) in PBS. After permeabilization, slides were washed twice for 5 min each time in 0.05% Triton X-100 in PBS. After this wash step, slides were flicked dry and a hydrophobic barrier was applied around tissue sections with an ImmEdge hydrophobic pen (H-4000, Vector Labs, Burlingame, CA, USA). A blocking solution consisting of 10% Normal goat serum (Cat# 10152-212, VWR, Radnor, PA, USA) and 0.1% Triton-X 100 in PBS was applied to each slide for 1 h. After this the blocking solution was replaced with fresh blocking solution containing primary antibodies and slides were incubated overnight at 4°C. On the following day primary antibody staining solution was discarded and slides were washed with 0.05% Triton-X 100 in PBS for 20 min × 4 times. A second blocking step was performed for 2 h at room temperature, before an overnight incubation with secondary antibodies diluted in blocking buffer at 4°C. On the following day slides were rinsed with PBS and stained for 15 min with DAPI (1 μg/ml) diluted in PBS. Finally, slides were washed 4× times with PBS and mounted using ProLong gold antifade mounting media (Cat# P36961, Thermo Fisher Scientific). All images were collected on an inverted Zeiss microscope and acquired with a sCMOS camera (Prime 95B, Teledyne Photometrics) under the control of micromanager 2.0 software. Primary antibodies used were Rabbit α mouse Gnat1 IgG (Ref: 55167-1-AP, Proteintech, Rosemont, IL, USA) or Rabbit α mouse GNAT2 IgG (Ref: PA5-22340, Thermo Fisher Scientific), both diluted to 1:250. The secondary antibody used was a goat α rabbit IgG Alexa Fluor 647 (Cat# A-21245, Thermo Fisher Scientific) diluted to 1:1000. The citrate buffer consisted of 10 mM sodium citrate (Cat# S279-500, Fisher Scientific, Hampton, NH, USA) with 0.05% tween-20 (Ref: P1379, Sigma-Aldrich), adjusted to pH 6.0. PBS consisted of 137 mM NaCl, 2.7 mM KCl, 10 mM Na_2_HPO_4_, and 1.8 mM KH_2_PO_4_ adjusted to a pH of 7.4. For all deparaffinization, rehydration, washing, permeabilization and DAPI staining steps, cells were placed on an orbital shaker and gently agitated. For all room temperature and overnight incubations, slides were placed in a sealed wet chamber to prevent solution evaporation and tissue desiccation.

### Genotyping

To confirm homozygous genotypes of DKO animals we digested tail tips from control and DKO mice and performed PCR reactions as outlined by Chen et al. (2020). To test for the presence of wildtype Gnat1 we used the following primers: *Gnat1* F (5′-CGAGTTCATTGCCATCATCTACG-3′) and *Gnat1* R (5′-ATACCCGAGTCCTTCCACAAGC-3′). To test for the presence of knockout Gnat1 we used the following primers: *TrKO F (5*′*-*GAGGATTGGGAAGACAATAGCAG-3′) and *TrKO* R (5′-CACCAGCACCATGTCGTAAG-3′). To test for the presence of the Gnat2^cpfl3^ mutation the following primers were used: *Gnat2* F (5′-GCAGGACGTGCTTCGATCCAG-3′) and *Gnat2* R (5′-CCTAGATGCTACAGCAGAAAGG-3′). For both Gnat1 and Gnat2 genotyping the same PCR protocol was as follows: denaturation at 95°C for 3 min, followed by 35 amplification cycles of 20 s at 95°C, 20 s at 60°C, and 30 s at 72°C, finalized with a 5-min extension at 72°C. DreamTaq Hot Start Green PCR Master Mix (2X, Cat# K9022, Thermo Fisher Scientific) was used for all PCR reactions. For Gnat2 genotyping, PCR product was first incubated at 37°C for 1 h with the restriction enzyme MseI (Cat# R0525S, New England BioLabs, Ipswich, MA, USA) in rCutSmart buffer (Cat# B6004S, New England BioLabs), then heat-inactivated at 65°C for 20 min before running it on a 2% agarose gel. MseI was used to target the unique restriction site that appears with the single nucleotide *cpfl3* mutation but is absent in the wildtype *Gnat2* gene.

## Results

### Double knockout VEP response dynamics differ markedly from those of wildtype animals

VEPs were elicited from control and double knockout mice across a dynamic range spanning 4 log units of stimulus intensity (Figure 2A), from 10^3^ photons/μm^2^ to 10^7^ photons/μm^2^ (532 nm light, 50 ms flashes). Even after averaging, we were unable to visually identify any positive waveforms for double knockout mice at a stimulus intensity below 3⋅10^5^ photons/μm^2^. Above this threshold VEPs in these animals grew rapidly with stimulus power, to magnitudes similar to those recorded in wildtype mice. When we compared the P1N1 amplitudes for VEPs elicited at the highest stimulus intensity used (10^7^ photons/μm^2^) we found no significant difference between DKOs and controls (Figure 2B). Visual inspection of VEPs at intensities that elicited positive VEPs in both control and DKO animals identified a marked delay in waveforms for the latter group (Figures 2C, D). We quantified this delay as latency of the P1 component and found that these latencies were significantly longer in DKO animals compared to controls for all intensities tested (Figure 2D). Interestingly, P1 latencies in DKO animals did not approach the values in control animals even at the highest stimulus intensity used. To compare the dynamic ranges of responses in control and DKO animals, we generated a scatterplot of normalized VEP power vs. stimulus intensity (Figure 2E). When we fit naka-rushton equations to these datasets, we found that the DKO stimulus response curve was right-shifted compared to the control curve by ∼2 log units (*I*_50_ = 7.87⋅10^5^ vs. 3⋅10^3^, Figure 2E). Additionally, in DKO mice the naka-rushton curve slope, a measure of response heterogeneity (Evans et al., 1993), was much steeper (0.817 vs. 0.156). We fit individual naka-rushton curves to the normalized power vs. stimulus illuminance datasets for each mouse and found that these two trends held, with mean DKO *I*_50_ and slope (Figure 2F) significantly larger than in controls. Together, these results demonstrate marked differences in VEP response dynamics between control and DKO mice.

### Double knockout VEPs are highly photobleachable

**FIGURE 3:**
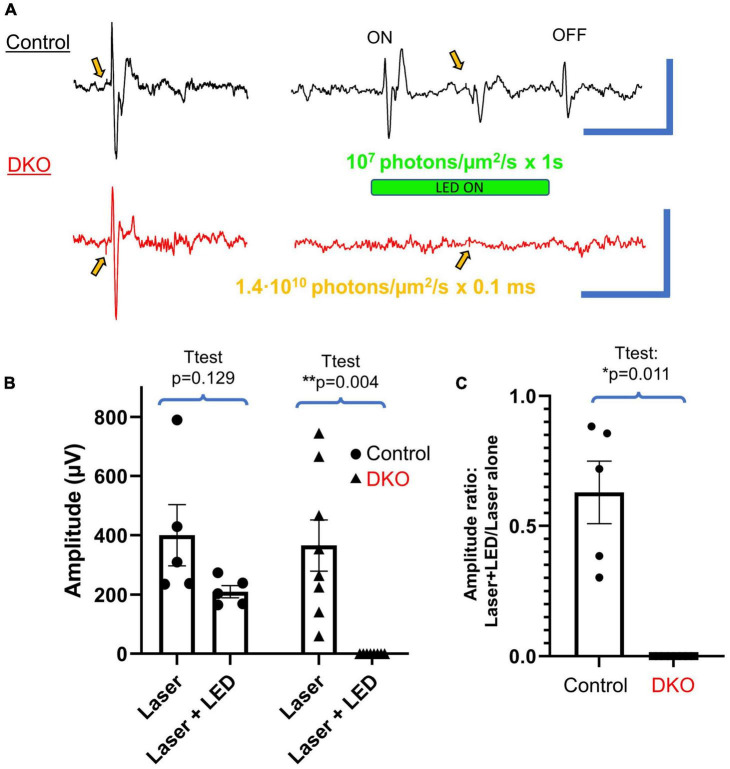
VEPs in DKO mice are highly photobleachable. **(A)** Typical VEPs elicited in control mice (top, black) and DKO mice (bottom, red) *via* 0.1 ms laser stimulation (520 nm, 1.4⋅10^6^ photons/μm^2^ delivered to the eye) alone (left) or with the same laser stimulation stacked on top of a 1 s LED stimulus (532 nm, 10^7^ photons/μm^2^ delivered to the eye, right). These saturating stimuli resulted in distinct LED-ON (ON), LED-OFF (OFF), and laser-induced responses in control mice, all of which are markedly absent in their DKO counterparts. Gold arrows indicate application of laser stimulus, usually accompanied by a distinguishable transient stimulus artifact. Scale bars represent 500 ms and 500 μV for both sets of traces. **(B)** Plots of average amplitudes ± SEM of the laser induced VEP alone vs. that measured during the saturating LED stimulus. Individual data points are shown for each control (black dots) and DKO (black triangles) mouse. Absolute amplitudes were not significantly reduced for control mice but were for DKO mice. **(C)** The same data as shown in panel **(B)**, but the ratio of VEP induced by laser stimulus + LED to that induced by laser alone. Control mice had significantly more preserved VEP responses compared to DKO mice (the latter having no visible response during the saturating stimuli). *Level of significant difference *via t*-test. *p < 0.05 and **p < 0.01.

To further characterize the VEP response in DKO animals, we performed an experiment to determine the saturability of their photoresponse. We first recorded VEPs in response to a short laser pulse, the photon flux of which exceeded the maximum photon flux of our LED by several orders of magnitude (1.4⋅10^10^ 520 nm vs. 10^7^ 532 nm photons/μm^2^/s, Figure 3A). We subsequently recorded VEPs elicited by a long (1 s) LED flash at maximum power (LED driver at 100%, ND0), intended to saturate the photoresponse, with the previously used laser stimulus applied halfway through the LED stimulus (Figure 3A). In wildtype animals this long LED stimulus produced a characteristic ON and OFF responses at the beginning and end of the stimulus, respectively. The laser stimulus applied on top of this saturating LED stimulus consistently produced an additional VEP. However, in DKO animals while robust VEPs were recorded in response to laser stimulation alone, the characteristic ON and laser-induced responses recorded in wildtype animals were markedly absent, or dramatically small in magnitude. Interestingly, no OFF response was recorded in DKO animals. The P1N1 amplitudes of the laser induced VEPs without and with the saturating LED stimulus are shown in Figure 3B. For control animals, while the average P1N1 amplitude was reduced when applied on top of the saturating LED, there was no significant difference (*t*-test, *p* = 0.129). However, as a laser stimulus failed to evoke any recognizable VEP in DKO animals when applied on top of the long LED stimulus, we found a significant reduction in P1N1 amplitude compared to the VEP elicited by laser stimulus alone (*t*-test, *p* = 0.004). By normalizing the amplitude of VEPs elicited by the laser+LED to the laser alone (Figure 3C) we compared the degree to which responses were spared in control vs. DKO mice, finding that responses were significantly less diminished in controls (*t*-test, *p* = 0.011). These data show that the mechanism mediating light sensitivity in DKO animals is saturable.

### Generation of robust VEPs in DKO animals occurs independent of rod or cone photoreception and major activation of retinal interneurons

**FIGURE 4:**
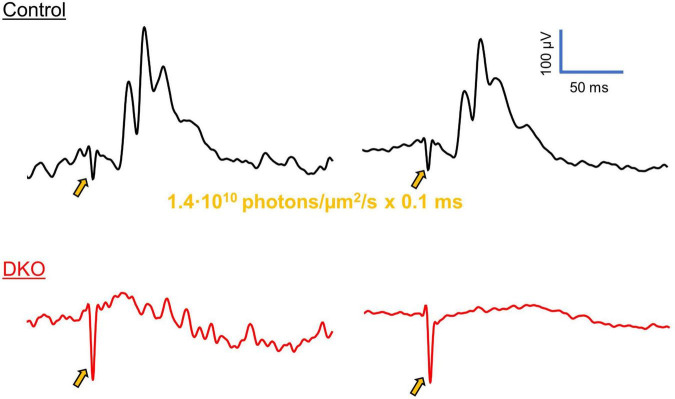
VEPs in DKO mice are independent of classical photoreception. Representative photopic ERG traces from control (**top**, black) and DKO (**bottom**, red) mice, elicited *via* 0.1 ms laser pulses (520 nm, 1.4⋅10^6^ photons/μm^2^ delivered to the eye). In control animals, ERG waveforms were clearly discernable from individual sweeps (left), or after averaging (right), with a small a-wave, robust b-wave, and oscillatory potentials. These characteristics reflect the activation of photoreceptors, ON bipolar cells and retinal interneurons, respectively. However, in the DKO mice none of these features were present, before (left) or after (right) averaging. Gold arrows indicate the onset of laser stimulus, accompanied by electrical artifacts. Similar ERG responses were recorded in *n* = 5 control and *n* = 5 DKO mice. The example waveforms shown here have been further cleaned with a 4-pole low-pass butterworth filter with a cutoff frequency of 200 hz.

To assess the retinal origin of VEPs in DKO mice, we attempted to record electroretinograms in control and DKO animals. In wildtype animals, ERGs were robust and easily discernable under light-adapted conditions (Figure 4), with a negative-going a-wave, positive-going b-wave, and oscillatory potentials. Importantly, these features were readily apparent from individual sweeps prior to averaging. When we attempted to record ERGs in DKO mice, we failed to elicit any of these characteristic waveforms.

**FIGURE 5:**
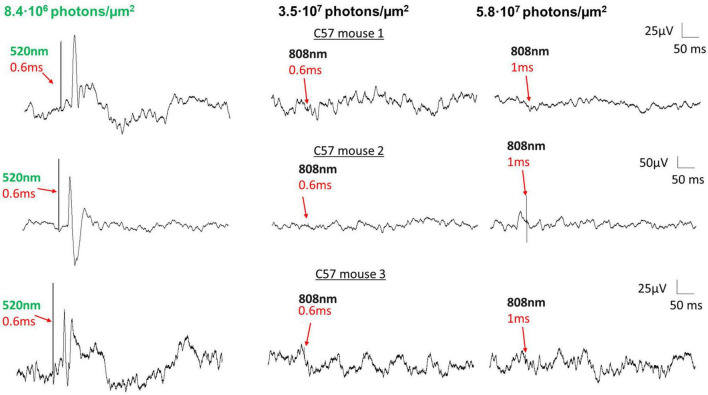
VEPs in DKO mice are not attributable to heat-induced optocapacitive excitation. Examples of VEPs recorded from three separate control mice with a 520 nm laser (8.4⋅10^6^ photons/μm^2^ delivered to the eye, **left**) and an 808 nm laser (3.5⋅10^7^ and 5.9⋅10^7^ photons/μm^2^ delivered to the eye, **middle**, **right**). For all three animals, VEP responses were robust upon 520 nm laser stimulation, but were markedly absent upon 808 nm laser stimulation, at equal or greater stimulus duration. Laser stimulus onsets and durations are as indicated. No positive VEP was recorded at any point when using the 808 nm laser as stimulus, suggesting that the localized tissue heating at this power level was insufficient to depolarize retinal neurons.

To preclude the possibility that the photoresponses we recorded in DKO mice are due to a non-specific heat-induced optocapacitive effect rather than a melanopsin-mediated signal, we performed an additional experiment. In wildtype mice, we first recorded VEPs with 520 nm laser stimuli (8.4⋅10^6^ photons/μm^2^) as a positive control (Figure 5, left). In the same animals, we subsequently switched the stimulus to an 808 nm laser of similar power and duration (3.5⋅10^7^ photons/μm^2^, Figure 5, middle). Because this wavelength lies far outside the absorption spectra of mouse photopigments (including melanopsin), it should not be able to activate photoreception *via* any of the established phototransductive mechanisms. In these experiments we failed to evoke any measurable VEP in wildtype mice with this stimulus, even when we increased the stimulus duration (5.8⋅10^7^ photons/μm^2^, Figure 5, right). This suggests that only certain wavelengths of light (i.e., those that are well-absorbed by melanopsin) are capable of generating visual perception in DKO mice.

**FIGURE 6:**
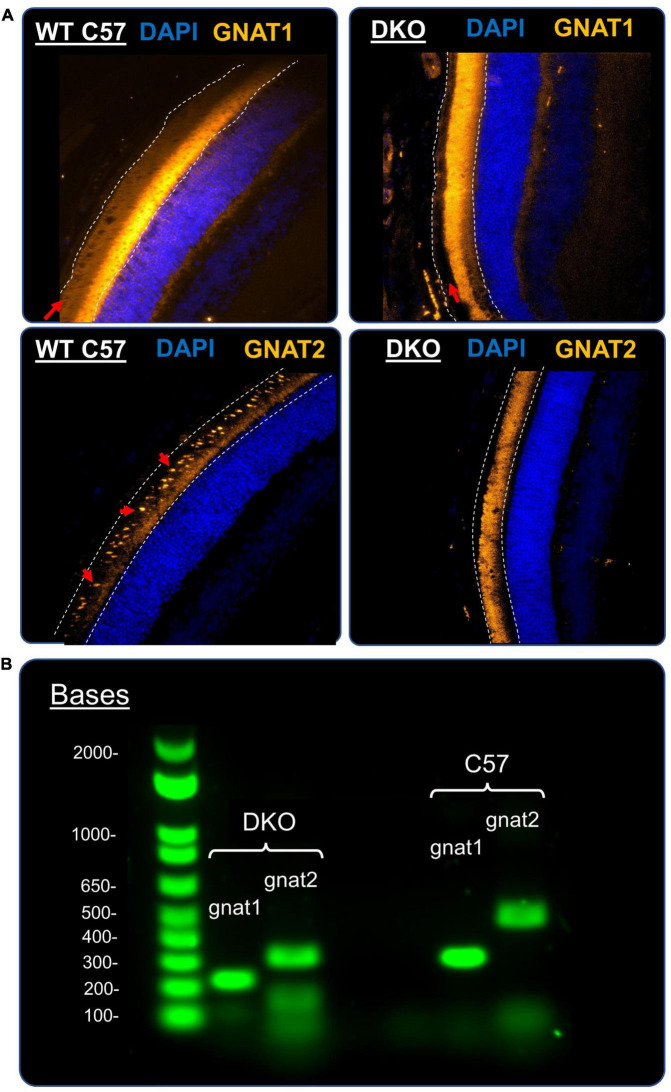
Confirmation of DKO phenotype and genotype. **(A)** Representative immunofluorescence images of retinal cross-sections stained for rod α-transducin (top, Gnat1, gold) and cone α-transducin (bottom, Gnat2, gold) in a control mouse (left) and a DKO mouse (right). White dashed lines delineate the photoreceptor outer segments layer. For Gnat1 staining (top) red arrows point to the presence of robust Gnat1 staining in the outer segments of rod photoreceptors. This staining is undetectable in DKO mice (top, right, red arrow), suggesting a successful knockout of Gnat1 expression. In both images, a large amount of autofluorescence is present at the border between the photoreceptor layer and outer nuclear layer, which was attributed to non-specific staining by the secondary antibody (as shown by a no-primary antibody control, data not shown). For Gnat2 staining (bottom), cone α-transducin expression was clearly present in the oval-shaped outer segments of cone photoreceptors (indicated by red arrows) in the control animals (bottom, left). This staining pattern was totally absent from our DKO retinal slices (bottom, right), suggesting that Gnat2 expression was significantly reduced. **(B)** Example 1% agarose gels showing the genotyping of a control mouse (left) and a DKO mouse (right), as described in our methods. For gnat1, the control and knockout bands were located at ∼300 and ∼200 bp, respectively. For Gnat2, the control and knockout bands were located at ∼480 and ∼300 bp, respectively. Our DKO animals showed the proper homozygous band pattern we would expect.

To further ensure that the DKO animals we employed in this study were properly lacking in rod and cone function, we immunostained wildtype and DKO retinal slices for the presence of rod or cone alpha-transducin protein. In a wildtype retina (Figure 6A, top left) staining for rod alpha-transducin (GNAT1, gold) showed robust expression in the outer segment of rod photoreceptors (red arrow). In DKO retina slices (Figure 6A, top right) one can note the complete absence of this protein in these outer segment (red arrow), suggesting successful knockout of GNAT1 expression. Similarly, in wildtype retinas cone alpha-transducin (Figure 6A, bottom left) shows up in the outer segments of the more sparse and rounded cone photoreceptors (GNAT2, gold, red arrows). The complete lack of a similar expression pattern in DKO retinas (Figure 6A, bottom right) suggests supremely diminished GNAT2 expression in our animal line, as has been identified previously (Chang et al., 2006; Chen et al., 2020). As further confirmation of our immunohistological findings, we genotyped wildtype and DKO animals for genetic modifications to the GNAT1 and GNAT2 gene loci. Figure 6B shows an example of this genotyping from a wildtype (C57BL/6) and DKO mouse. The wildtype band for gnat1 is at ∼300 bp compared to ∼200 bp in the DKO animals. The wildtype band for gnat2 is at ∼480 bp compared to ∼300 bp in the DKO animals. The clear separation of these bands and the absence of wildtype bands in the DKO mice confirms the successful knockout of these genes in our animal model.

## Discussion

This study builds on previous work that has attempted to discern the contribution of melanopsin phototransduction to pattern-forming vision. As previously stated, this task is highly non-trivial, as no single experimental approach to elucidate melanopsin’s contribution is without its caveats. Genetic knockout models that disrupt key components of rod, cone, or melanopsin-specific phototransduction, or target specific cell types for apoptosis, have been employed to this research question with great success. However, conditional knockout models such as those utilizing a *cre* system suffer from a degree of “leakiness,” rarely achieving complete elimination of targeted proteins or cell types. Inducible knockout models that are useful for preserving a gene or cell type’s function during development also suffer from this drawback. This leakiness makes it difficult to disregard possible contributions of residual rod, cone, or melanopsin-mediated activity to visual perception under conditions where these studies assume one or two of these sources have been completely eliminated. The main advantage to global knockout models is the absence of such leakiness, at the cost of possible developmental deficits as has been suggested to occur in Opn4^–/–^ mice (Tufford et al., 2018). Retinal degeneration models that rely on massive loss of rod and cone photoreceptors to isolate melanopsin activity also suffer from the potential for small pockets of surviving photoreceptors to confound results. Additionally, there is some evidence that retinal circuitry can be dramatically remodeled in such disease states (Marc et al., 2007), complicating interpretations of results. More atypical approaches to examine melanopsin’s role in pattern-forming vision include chemogenetic and silent-substitution strategies, which have the advantage of probing melanopsin without disrupting any of the photoreceptive systems. However, the chemogenetic approach cannot examine the complex kinetics and activation dynamics of melanopsin that occur during photic stimulation. The use of silent-substitution methods, which utilize complex light stimuli to selectively activate one photoreceptor class while causing no change in the activity of others would appear to be the most ideal way to examine this question. However, certain experimental complexities inherent to the technique and the visual system itself make such selectivity for a single photoreceptive class a difficult goal to achieve (Spitschan and Woelders, 2018; Conus and Geiser, 2020). Recent results also indicate that even when an ideal “silencing” of the voltage response of cone photoreceptors is achieved, changes in their glutamatergic output still occur (Kamar et al., 2019). Suffice to say, it is difficult to design a single experiment that could adequately address all of the disadvantages of the varied strategies that have been utilized to date.

Here we present results from a further refinement of the global knockout approach, using a genetic model purported to have no residual rod or cone activity under certain conditions while otherwise preserving retinal structure and connectivity. Our data shows that robust VEPs, representing the light-induced spiking of large numbers of cortical V1 neurons, can be induced *via* melanopsin signaling. This finding conforms with previous studies demonstrating large levels of melanopsin-induced activity in the primary visual pathway, though these other studies utilized complementary techniques such as dLGN recordings, retinal multielectrode arrays, and *cFos* immunofluorescence. Our findings do not presume to offer a final word on this topic; rather, they yield additional support to the notion that melanopsin signaling plays an integral role in conscious visual perception that requires further study.

### VEP characteristics are consistent with a melanopsin-mediated photoresponse

VEPs elicited in our DKO mice differed markedly from those recorded from their wildtype counterparts in their absolute threshold, kinetics and saturability (Figure 2). The stimulus threshold for melanopsin activation is generally reported to be on the order of 10^4^–10^6^ photons/μm^2^/s (Estevez et al., 2012; Davis et al., 2015; Schroeder et al., 2018), depending on stimulus wavelength and duration, as well as ambient light conditions and retinal adaptation state (Davis et al., 2015). Zhao et al. (2014) reported a slightly lower threshold (10^3^ photons/μm^2^/s) for melanopsin activation but suggested that in general melanopsin’s threshold is ∼2 log units greater than that of cones. In our experiments, the threshold stimulus intensity for obtaining a positive VEP signal in DKO mice was approximately 7.1⋅10^8^ photons/μm^2^/s. In wildtype mice this threshold was closer to 7.1⋅10^6^ photons/μm^2^/s, about 2 log units dimmer. These absolute intensities may seem to be on the higher end; however, most studies performed on ipRGCs use stimuli that last on the order of seconds to minutes, compared to the 50 ms flashes used here. If one calculates the total photon flux of our DKO threshold stimulus, 3⋅10^5^ photons/μm^2^, and takes into account the light-adapted state of our animals, it is consistent with melanopsin activation over that of rod or cone photopigments.

We found the VEP latency to be significantly longer in DKO mice compared to wildtypes (Figures 2C, D), consistent with the sluggish kinetics (Zhao et al., 2014) of melanopsin phototransduction versus that of rods and cones. The delayed activation and prolonged depolarization of ipRGCs in response to light also explains our failure to elicit VEPs in DKO mice with our saturating stacked stimuli (Figure 3), as the short interval between LED and laser onsets, as well as the 5 s inter-sweep period, are not long enough for the recovery of the intrinsic photoresponse.

### Residual photoreceptor activity is not responsible for light-evoked cortical potentials in DKO mice

One potential confounder of this present study is the concern that the mouse model employed in our experiments still retains a small degree of photosensitivity *via* the rod or cone pathway. This photosensitivity is evidence by the existence of recordable electroretinogram a- and b- waves, as well as measurable photocurrents in isolated photoreceptors, at least under dark-adapted conditions (Allen et al., 2010; Chen et al., 2020). Although photoreceptors of DKO animals appear vastly less sensitive to light than their wildtype counterparts, it seems that their mutant form of cone α-transducin can still activate the phototransductive pathway given a stimulus of sufficient intensity (Chen et al., 2020). To circumvent this limitation, all of the experiments included in this study were performed under light-adapted conditions, at ambient illumination that seems to shut-down this residual activity as demonstrated by the lack of a photopic electroretinogram (Figures 3B, 4; Chang et al., 2006). The absence of a photopic ERG waveform in DKO mice suggests the origin of their positive VEP signals to be the direct depolarization of ganglion cells (bypassing photoreceptor → bipolar cell → ganglion cell circuits), in this case by the photopigment melanopsin. Further proof that cone photoreceptors are not responsible for our witnessed VEPs is the lack of a discernable OFF response to our sustained LED stimuli (Figure 3) in DKO animals compared to controls. If residual cone activity was the mechanism responsible for preserved cortical activity, one would expect both the ON and OFF retinal pathways to be active.

### Early receptor potential or optocapacitance are unlikely mechanisms for witnessed cortical potentials

When using light stimuli at the intensities employed in this study, one must be careful in assigning the origin of a photoresponse to either of the classical phototransductive pathways. Two obvious mechanisms that bypass the canonical pathway are the early receptor potential and optocapacitive excitation. The early receptor potential is a transient hyperpolarization that occurs in both rod and cone photoreceptors upon stimulation with a sufficiently bright stimulus (Brown et al., 1965). It can be attributed to the direct displacement of charge in rhodopsin and cone opsin molecules during the conformational changes that accompany photoisomerization (Cone, 1967; Woodruff et al., 2004). Importantly, this transient hyperpolarization occurs independent of the signal amplification imparted by the phototransductive cascade and could conceivably be responsible for the light responses recorded in our double knockout mice. However, this mechanism suffers from an inherent limitation that makes it an unlikely candidate for our observed responses: in order to create a hyperpolarization of any noticeable magnitude, a significant portion of photopigment needs to be bleached (Woodruff et al., 2004), and the percent of unbleached photopigment that remains upon each subsequent stimulus makes this process rapidly exhaustible. Furthermore, when we recorded ERGs from DKO mice we failed to elicit any discernable b-wave (Figure 4), suggesting that regardless of any transient photoreceptor hyperpolarization that could have occurred, retinal bipolar cells were not activated to any appreciable degree.

Optocapacitive excitation is a mechanism by which rapid highly localized changes in temperature close to a cell membrane, as can occur during laser stimulation, induces neuronal depolarization (Shapiro et al., 2012; Carvalho-de-Souza et al., 2018; Pinto et al., 2022). Because this phenomenon can activate any cell, independent of the presence of phototransductive machinery, it is conceivable that the light stimuli employed here could bypass retinal first and second-order neurons to depolarize ganglion cells directly, regardless of the presence of melanopsin. To rule this possibility out we attempted to record VEPs in wildtype mice with an 808-nm laser (Figure 5). While VEPs were robust in these animals in response to stimulation with a 520 nm green laser, no evoked potentials were seen when stimulating with 808 nm stimuli, even when the stimulus duration (and thus total energy) was increased beyond that of our 520 nm stimuli. This result confirms that localized heating and optocapacitive depolarization is not the mechanism responsible for generating the cortical potentials reported here.

### Melanopsin-driven VEPs are just as robust as wildtype VEPs at saturating stimuli

One major result that we found to be very surprising was the similarity in the maximal magnitudes of recorded VEPs in control and DKO mice (Figure 2B), suggesting the activation of similar numbers of cortical neurons in V1. This is interesting because ipRGCs only represent ∼4–5% of all retinal ganglion cells (Hughes et al., 2013; LeGates et al., 2014), and some classes of ipRGCs do not majorly project to vision-forming nuclei such as the dLGN (Monavarfeshani et al., 2017; Aranda and Schmidt, 2021). This result can be partially explained by recent findings demonstrating an overrepresentation of dLGN innervation by the M4 class of ipRGCs (Román Rosón et al., 2019). Additionally, this study highlighted a previously unappreciated degree of convergence by retinal afferents onto dLGN neurons, showing that many dLGN neurons actually receive mixed input from a diversity of different ganglion cell populations. It is possible that in the absence of input from other populations of non-ipRGC ganglion cells in DKO animals, homeostatic mechanisms could cause a larger proportion of dLGN neurons to fire in response to input from the ipRGC populations that maintain photosensitivity.

Another possible mechanism is the formation of abnormal activation networks between ipRGCs and other ganglion cell populations. Eleftheriou et al. (2020) showed in dystrophic retinas that melanopsin signaling in ipRGCs was likely responsible for the activation of widespread spiking in non-ipRGC ganglion cells, potentially *via* gap-junctional coupling. This widespread activity could not be reproduced in wildtype animals, or in retinal dystrophic animals lacking melanopsin. Although retinal morphology and connectivity is thought to remain largely intact in this animal model (Chang et al., 2006; Ronning et al., 2018; Chen et al., 2020) some retinal remodeling similar to that witnessed in dystrophic Rd1 mice might occur that could produce similar widespread spiking in non-ipRGCs upon melanopsin activation.

### Implications for melanopsin’s contribution to vision

The results presented here indicate an ability for melanopsin to produce robust cortical responses in the absence of major rod or cone input. A more difficult issue to resolve is whether this activity is actually capable of representing a useful visual percept. A recent study examining contrast sensitivity in various mouse lines found evidence for such a percept in the same animal model that we employed here (Pasquale et al., 2020; Figure 3B). Through the use of an optomotor assay, Pasquale et al. (2020) identified an ability for these animals to track a moving grating sinusoid, although only at fairly bright stimulus intensities (∼50,000 photoisomerizations/rod/s). Importantly, this ability was still dramatically impaired compared to that of wildtype animals. Taking this study into consideration, our findings would suggest the possibility that at even brighter stimulus intensities the performance of DKO mice could begin to rival that of wildtype animals, at least in the perception of contrast. However, the inherent limitations of melanopsin phototransduction kinetics and its selective expression in only certain ganglion cell populations ultimately constrain its utility as a sole photoreceptive pigment. A recent study in humans found that by using silent-substitution methods, spatial patterns designed to selectively activate melanopsin over cones could indeed be perceived by participants (Allen et al., 2019). However, as expected these patterns were only distinguishable at low spatial and temporal frequencies. If melanopsin was sufficient for visual function under bright enough conditions, one would expect to have identified this phenomenon in certain individuals affected by various retinal pathologies, some of which largely spare ipRGC populations despite outer retinal degeneration (Semo et al., 2003; Cui et al., 2015; Tran et al., 2019; Procyk et al., 2022). Although there is some evidence for residual melanopsin-mediated activity in such individuals (Castaldi et al., 2019), it is unclear whether any of this activity can even be perceived. It seems more likely that melanopsin, rather than directly extending the dynamic range of the retina to extremely bright intensities, instead primarily mediates a range of functions that serve to accentuate and fine-tune certain aspects of pattern-forming vision. Examinations of human subjects have begun to identify roles for melanopsin in specific percepts, such as absolute brightness (Yamakawa et al., 2019) and color constancy (Zele et al., 2018). At a more fundamental level, there is some evidence that M1 ipRGCs are involved in the regulation of dopaminergic amacrine cells (Prigge et al., 2016; Munteanu et al., 2018), which in turn are believed to underlie retinal light adaptation (Roy and Field, 2019). Through this signaling axis alone, melanopsin could initiate profound changes to rod- and cone-mediated vision prior to any post-retinal processing. Perhaps most intriguingly of all, Sonoda et al. (2018) showed that melanopsin is capable of enhancing the contrast sensitivity of M4 ipRGCs across a wider range of light intensities than had previously been considered possible, all the way from scotopic to photopic conditions. This means that the impact of melanopsin on pattern-forming vision is not constrained by the traditionally accepted high thresholds for melanopsin activation. Ultimately, the topic of melanopsin’s contributions to pattern-forming vision is one that defies simple explanation and will require further study.

## Conclusion

This study builds on previous work that has and continues to identify roles for ipRGCs in pattern-vision. Here, using a mouse model under conditions in which the classical pathways for photoreception are inactive, we showed that bright light stimuli could still evoke robust activity in primary visual cortex, likely through the direct excitation of ipRGCs. Although such visual activity is probably constrained by certain spatial and kinetic limitations, ipRGCs could potentially serve as a useful visual substrate in a certain population of blind individuals, without the need for more invasive and costly interventions.

## Data availability statement

The original contributions presented in this study are included in the article/supplementary material, further inquiries can be directed to the corresponding author.

## Ethics statement

The animal study was reviewed and approved by The University of Arizona Institutional Animal Care and Use Committee (IACUC).

## Author contributions

JC-d-S, SH, and MF conceived the research. MF performed the electrophysiological recordings research. HV performed the mice genotyping methods. MF analyzed the data with inputs from JC-d-S. MF wrote the manuscript and received edits and comments from JC-d-S and HV. All authors contributed to the article and approved the submitted version.
